# Optimized beam shaping assembly for a 2.1-MeV proton-accelerator-based neutron source for boron neutron capture therapy

**DOI:** 10.1038/s41598-021-87305-9

**Published:** 2021-04-07

**Authors:** Pablo Torres-Sánchez, Ignacio Porras, Nataliya Ramos-Chernenko, Fernando Arias de Saavedra, Javier Praena

**Affiliations:** Departamento de Física Atómica, Molecular y Nuclear, Facultad de Ciencias, Universidad de Granada, 18071 Granada, Spain

**Keywords:** Biotechnology, Radiotherapy, Materials for devices

## Abstract

Boron Neutron Capture Therapy (BNCT) is facing a new era where different projects based on accelerators instead of reactors are under development. The new facilities can be placed at hospitals and will increase the number of clinical trials. The therapeutic effect of BNCT can be improved if a optimized epithermal neutron spectrum is obtained, for which the beam shape assembly is a key ingredient. In this paper we propose an optimal beam shaping assembly suited for an affordable low energy accelerator. The beam obtained with the device proposed accomplishes all the IAEA recommendations for proton energies between 2.0 and 2.1 MeV. In addition, there is an overall improvement of the figures of merit with respect to BNCT facilities and previous proposals of new accelerator-based facilities.

## Introduction

Boron Neutron Capture Therapy (BNCT) is an experimental form of radiotherapy which is selective at the cellular level. In recent years BNCT is back in limelight due to the most recent successful clinical trials for very malignant diseases like brain tumors and recurrent head and neck cancers^1^. In addition, with the advances in accelerator technologies, now it is possible to obtain neutron beams suitable for BNCT in facilities that can be built inside hospitals, and different projects had been started for this purpose throughout the world^2,3^.

The first accelerator-based BNCT facility suitable for clinical trials (C-BENS) has been developed in the Kyoto Research Reactor Institute with a 30-MeV cyclotron of Sumitomo Heavy Industries, and clinical trials have been recently started^4^. This pioneering facility may boost the development of new centers. Most of the other projects make use of lower energy proton beams on targets of $\mathrm {^7Li}$ or $\mathrm {^9Be}$, in the aim of producing lower energy neutrons, thus requiring less moderation in order to reach the optimal energies for BNCT, which are known as epithermal energies (up to 10 keV).

The International Atomic Energy Agency (IAEA), in the Technical Document of 1990^5^ establishes some recommendations for the beam quality for BNCT treatments. These recommendations limit the dose due to fast neutrons and gamma contamination, as well as the thermal neutron flux below certain upper bounds, provide a condition on the divergence of the beam and a lower bound for the epithermal neutron flux. Fulfilling all these recommendations from an accelerator-based neutron source is a challenging task. The moderation of the high-energy neutrons produced at the target to the epithermal energies for reducing the fast neutron dose increases the thermal neutron ratio and decreases the total neutron flux, so the selection of materials and their dimensions is critical for achieving all the recommendations.

In this work, within the frame of the NeMeSis Project^6^, we present a design of a beam shaping assembly (BSA) that accomplishes all these recommendations and which produces a very well defined beam for BNCT with low proton energies (between 2.0 and 2.1 MeV) on a Li target. This is a very interesting possibility as the accelerator required can be substantially cheaper than those required for other options at higher energies. In addition, the in-phantom figures of merit, which are essential for the suitability as a therapeutic beam for deep seated tumors, obtained from our design compare very well with other proposals published in the literature.

This paper is structured as follows. “Results and discussion” section shows the results from the optimization of the BSA and discusses the beam quality against other facilities of its kind. The conclusions derived from this research are highlighted in “Conclusions” section. “Methods” section describes the choice of target and the simulations carried out for the optimization of the BSA, together with the set beam parameters and of figures of merit used in the beam quality assessment.

## Results and discussion

The design of the geometry and the material choice was established following a optimization to meet the IAEA recommendations for BNCT^5^. In addition, it was paramount that the neutron spectrum peaked around 2–9 keV, matching the most adequate neutron energies for reaching deep-seated tumors.

### BSA design

The use of low energy proton beams profit from a less extended high energy tail in the neutron spectrum. In these conditions, $\mathrm {^7Li}$ is a best suited target option, provided its less energetic reaction yield compared to $\mathrm {^9Be}$ or spallation targets as Ta, W or Pb. Indeed, the pursuit to stick to low energy proton beams imposes a further dim of neutron production. This aspect has to be compensated with a higher intensity particle accelerator. In the following, we assume a 30 mA proton beam onto a metallic Li target. The proton energy range from the threshold to 2.3 MeV was studied. This choice limits the use of the broad 2.25 MeV resonance but also avoids the population of the first excited state of $\mathrm {^7Be}$ at 431 keV, whose threshold is found at 2.373 MeV, even considering that the neutron yield from this channel is less than 10% below 5 MeV^7^. The aim of this work is to explore for the first time the possibility of producing a neutron beam suitable for BNCT at the lowest energy possible, with the aim of strongly restricting the high energy tail due to kinematics. For example, for protons at 2.1 MeV onto $\mathrm {^7Li}$ , no neutrons are produced above 350 keV, which can reduce the effects on normal tissues due to the fast neutrons. Working at that low energies also avoids a region (400 keV to 1 MeV) where, for the inelastic scattering of $^{19}\hbox {F}$ (a main component of the moderator), there are discrepancies between experimental and evaluated data for the cross sections. Also the inelastic scattering with the Mg isotopes is avoided, as the first excited state of their isotopes occurs at 585 keV.

In the reaction between the protons below 2.3 MeV and the $\mathrm {^{7}Li}$, neutrons of up to few hundreds of keV are generated in the forward direction. Therefore, a neutron moderator with low absorption at these energies is fundamental for achieving an intense enough neutron beam for treatments. $\mathrm {MgF_2}$ has been considered extensively for the moderation of neutrons from accelerator-based sources^8^. The reason is the low capture cross-sections and complementary location of resonances of both fluorine and the magnesium isotopes. In our case, special emphasis comes near 100 keV, around the mean energy of neutrons produced at the $\mathrm {^{7}Li}$ source at 2.1 MeV. In that region, a pair of resonances partially overlap, giving a broad range for continuous neutron energy reduction. The $\mathrm {MgF_2}$ also surrounds the $\mathrm {^{7}Li}$ source in the backward direction in a cylindrical shape in order to recover medium-energy back-propagated neutrons. This structure is fully covered by a Pb shield that works as a neutron reflector. In the forward direction, the neutron moderation is completed by means of an Al layer, that in addition effectively filters the remaining neutrons by its resonances at 35 and 87.5 keV. Then, a ${{\mathrm {LiF}}}$ sheet is placed to reduce the over-moderated thermal neutrons so as to increase the beam penetrability. Finally, a Bi layer efficiently attenuates the gamma radiation generated in the previous steps, without a manifest effect in the neutron flux unlike Pb, profiting from its lower elastic cross-section. For making the design more realistic, a 1 mm layer of water for cooling the lithium target^9^ and an air gap of 1 mm around the accelerator tube have been considered for the simulations.

**Figure 1:**
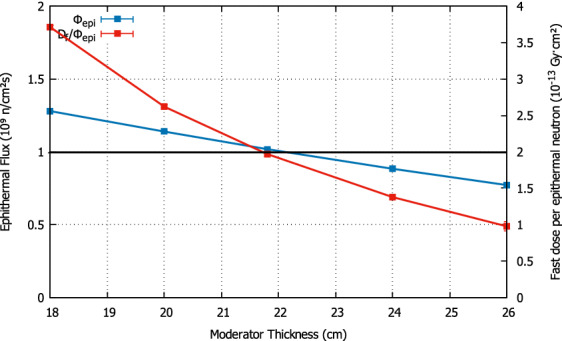
Optimization of the moderator thickness. The optimal value is found by keeping the epithermal flux (blue line) above the IAEA minimum recommendation (black line), while the fast dose per epithermal flux (red line) is below the IAEA maximum value recommended (same black line). This happens for a thickness of 21.8 cm.

**Figure 2:**
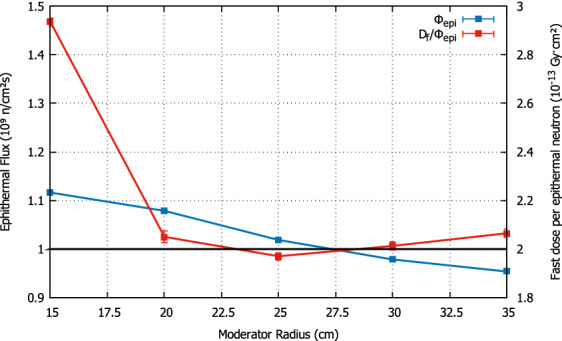
Optimization of the moderator radius for the optimal thickness. The optimal value is found by keeping the epithermal flux (blue line) as high as possible while the fast dose per epithermal flux (red line) is minimized, which happens at a radius of 25 cm.

The dimensions of the elements in the BSA have been chosen optimizing the figures of merit of the in-air IAEA recommendations, by means of a compromise between making the epithermal flux as high as possible while keeping the fast dose below the recommended value. This is illustrated as an example in Figs. 1 and 2 for the most critical dimensions (moderator length and radius).

Once the neutron spectrum achieved is adequate, it is key to shape and collimate the beam to achieve a satisfactory divergence and fairy low neutron and gamma contamination off-beam. The beam shaping and focusing is managed by means of a tapered geometry towards the beam aperture. Pb is used to this aim, benefiting from its reflector behavior and low moderating effect. For the off-beam contamination suppression, lithiated polyethylene blocks, combined with ${\mathrm {LiF}}$ and ${\mathrm {Pb}}$ layers accomplish this goal.

**Figure 3:**
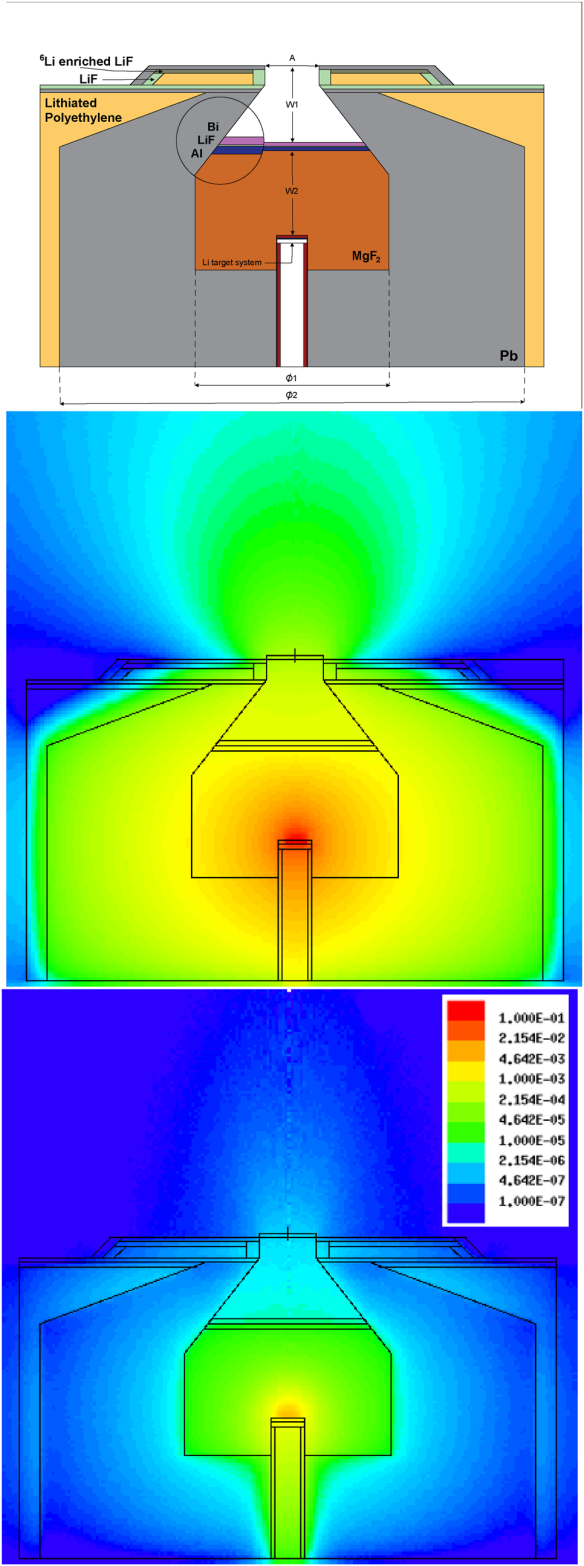
*Upper panel*: Layout of the BSA design, including general sizes and material specification. *Middle* and *lower panel*: Neutron and gamma radiation flux distribution throughout the BSA. The color key illustrates the neutron and photon intensity relative to the total neutrons generated in the target.

The optimization of the BSA for the choice of 2.1 MeV proton energy, brought in a selection of dimensions that meet the IAEA recommendations and serve adequately for a BNCT treatment. The direct along-the-axis thickness of the $\mathrm {MgF_2}$ core moderator (W2) is 21.8 cm. The total diameter of the moderator (ø1) is 50 cm. The successive Al, LiF and Bi filters account for 1.0 cm, 0.2 cm and 1.0 cm each, respectively. The surrounding Pb reflector has an outer diameter (ø2) of 120 cm. The distance from the Bi layer and the neutron beam aperture (W1) is 19.8 cm, and the beam aperture diameter (A) is 14 cm. All materials have been considered as pure, with a density of 3.148 $\mathrm {g/cm^3}$ for $\mathrm {MgF_2}$, 2.365 $\mathrm {g/cm^3}$ for ${\mathrm {LiF}}$ and 1.06 $\mathrm {g/cm^3}$ for lithiated polyethylene. A 100% enrichment was assumed for $\mathrm {^{6}LiF}$, and a 7.56% mass content of $\mathrm {^{6}Li}$ in lithiated polyethylene. Figure 3 (*upper panel*) illustrates the BSA final configuration.

**Figure 4:**
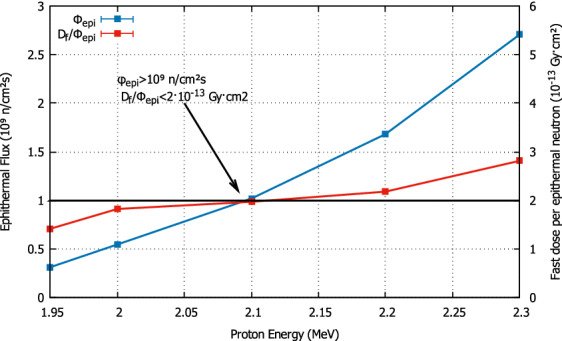
Detail of the Epithermal Flux and Fast Dose as a function of the proton energy incident on the $\mathrm {^7Li}$ target, compared with the IAEA recommendation figures. The value of 2.1 MeV is marked as both the epithermal flux and the fast dose are attained above and below their criterion, respectively.

Concerning the proton energy for the neutron production onto $\mathrm {^7Li}$, the optimal energy was found to be 2.1 MeV. Table 1 shows the in-air beam parameters that are achieved with our configuration of the BSA at different energies in the range 1.95 to 2.3 MeV. In addition, Fig. 4 explicitly shows the dependence of epithermal flux and fast dose with proton energy, pointing that 2.1 MeV corresponds to the energy of the best compromise among both criteria.

**Table 1:** Free-air figures of merit for the conformed beam, for different incident proton energies from 1.95 to 2.3 MeV, compared to with the IAEA recommendations.

	IAEA recommendation	Proton energy (MeV)				
		1.95	2.0	2.1	2.2	2.3
$\mathrm {\phi _{epi} \, (n/cm^2 \; s)}$	$>5 \cdot 10^8$	$3.124 \cdot 10^8$	$5.459 \cdot 10^8$	$1.019 \cdot 10^9$	$1.681 \cdot 10^9$	$2.706 \cdot 10^9$
$\mathrm {\phi _{th}/\phi _{epi}}$	$< 0.05$	0.0385	0.0378	0.0372	0.0364	0.0349
$\mathrm {J_n/\phi _n}$	$>0.7$	0.7138	0.7128	0.7120	0.7109	0.7099
$\mathrm {D_{fast}/\phi _{epi} \, (Gy \; cm^2)}$	$< 2 \cdot 10^{-13}$	$1.42 \cdot 10^{-13}$	$1.82 \cdot 10^{-13}$	$1.97 \cdot 10^{-13}$	$2.18 \cdot 10^{-13}$	$2.81 \cdot 10^{-13}$
$\mathrm {D_\gamma /\phi _{epi} \, (Gy \; cm^2)}$	$< 2 \cdot 10^{-13}$	$1.05 \cdot 10^{-13}$	$1.01 \cdot 10^{-13}$	$0.99 \cdot 10^{-13}$	$0.95 \cdot 10^{-13}$	$0.96 \cdot 10^{-13}$

The figures for $\mathrm {E_p} = 2.1$ MeV and $\mathrm {E_p} = 2.0$ MeV are the only ones that satisfy all criteria at once.

### In-air IAEA beam parameters

In Table 1 we compare the in free-air parameters with the IAEA recommendations^5^. The neutron flux, divergence, and contamination from gammas, thermal and fast neutrons were evaluated at the beam aperture in free air conditions. It is clear from the results that all IAEA recommendations are fulfilled at 2.1 MeV, while only the epithermal flux is below $\mathrm {10^{9}\, n/cm^{2}/s}$ at 2.0 MeV. Despite this, it is above the minimum recommendation of $\mathrm {5\cdot 10^{8}\, n/cm^{2}/s}$. This indicates that energies below 2.1 MeV down to 2.0 MeV are also valid for use with this BSA. With this geometry, more energetic proton beams would result in a higher energy neutron yield, specially considering the close reaction resonance at 2.25 MeV. That would no longer compensate the fast neutron dose contribution. On the other hand, proton beams below 2.0 MeV could not sustain a neutron flux high enough to operate the beam for treatments.

Figure 3 illustrates the neutron (*middle panel*) and gamma (*lower panel*) radiation flux distribution throughout the BSA, in free air conditions.

Apart from the epithermal flux, the thermal and current to flux ratios are well managed, found adequately within the recommendations. The gamma radiation dose contamination is well attained below the safe margins, even including the contributions from the $\mathrm {^{7}Li}$ source and the Al activation. The fast neutron dose contamination is properly settled just at the limit of the IAEA when defining the epithermal range from 0.5 eV to 10 keV. This gives rise to a more detailed remark on this point. This recommendation for the fast neutron component is currently under revision, as there has been suggested that neutrons slightly above 10 keV can also be useful for therapy^21^. Table 2 shows the performance of the BSA both at 2.0 and 2.1 MeV, each considering an epithermal upper limit at 10 and 20 keV. If one considers the epithermal range up to 20 keV, then the fast neutron dose reduces down to less than a half. Only 51% of the fast neutron flux corresponds to neutrons above 20 keV. Neutrons above 40 keV are only 32% of the total fast neutron flux. This is due to the fact that the starting neutron spectrum at the source has a much shorter energy tail than in usual beams. This underlines the advantageous properties of the beam in terms of contamination. For completeness, the results at a proton energy of 2.0 MeV turn out to be slightly more adequate in terms of beam contamination, with the drawback of a less intense neutron flux. Considering all these, in the following, all the remaining results will be given for the BSA solution at a proton energy of 2.1 MeV.

**Table 2:** In-air beam parameters compared to the IAEA recommendations, for proton accelerator energies of 2.0 and 2.1 MeV. The results considering the epithermal upper limit at 10 and 20 keV are included.

	IAEA recommendation	Our proposal (2.0 MeV)		Our proposal (2.1 MeV)	
		Epith. limits	Epith. limits	Epith. limits	Epith. limits
		0.5 eV–10 keV	0.5 eV–20 keV	0.5 eV–10 keV	0.5 eV–20 keV
$\mathrm {\phi _{epi} \, (n/cm^2 \; s)}$	$>5 \cdot 10^8$	$5.459 \cdot 10^8$	$5.783 \cdot 10^8$	$1.019 \cdot 10^9$	$1.081 \cdot 10^9$
$\mathrm {\phi _{th}/\phi _{epi}}$	$< 0.05$	0.0378	0.0357	0.0372	0.0351
$\mathrm {J_n/\phi _n}$	$>0.7$	0.7128	0.7130	0.7120	0.7119
$\mathrm {D_{fast}/\phi _{epi} \, (Gy\; cm^2)}$	$< 2 \cdot 10^{-13}$	$1.82 \cdot 10^{-13}$	$0.94 \cdot 10^{-13}$	$1.97 \cdot 10^{-13}$	$1.09 \cdot 10^{-13}$
$\mathrm {D_\gamma /\phi _{epi} \, (Gy \; cm^2)}$	$< 2 \cdot 10^{-13}$	$1.01 \cdot 10^{-13}$	$0.92 \cdot 10^{-13}$	$0.99 \cdot 10^{-13}$	$0.97 \cdot 10^{-13}$

When comparing the results from this BSA and previous designs for BNCT, one has to mention the features of previous and current reactor-based BNCT facilities, such as FiR-1 in Finland^10,11^, KURRI in Japan^12^ or currently THOR in Taiwan^13^. KURRI in epithermal mode and THOR share high fast neutron contamination (6.1 and 2.8, in units of $10^{-13}$ $\text{Gy} \; \text{cm}^2$ per epithermal neutron), and also a high thermal neutron ratio (0.12) in the case of THOR. However, they had convenient beam intensity ($9.1\cdot 10^8$ and $1.7\cdot 10^9$ $\text{n}/\text{cm}^2 \; \text{s}$ at KURRI and THOR, respectively) and divergence (0.81 in the case of THOR). Concerning accelerator-based facilities (AB-BNCT), C-BENS is the first facility already in operation^14^. It has a high intensity ($1.2\cdot 10^9$ $\text{n}/\text{cm}^2 \; \text{s}$) and low thermal neutron contamination (0.04), yet the fast neutron dose exceeds the maximum ($5.8\cdot 10^{-13}$ $\text{Gy} \; \text{cm}^2$) even considering an extended epithermal range to 40 keV^14^. Other projects and designs achieve desirable beam intensities and low thermal contamination, though most of them suffer from high fast neutron contamination^15–17^, while others do not achieve a tight collimation and low divergence^18,19^. A design in Italy achieves high epithermal flux, but doses from fast neutrons and gamma radiation remain higher than recommendations^20^.

### Neutron spectrum and lateral out-of-field flux profiles

**Figure 5:**
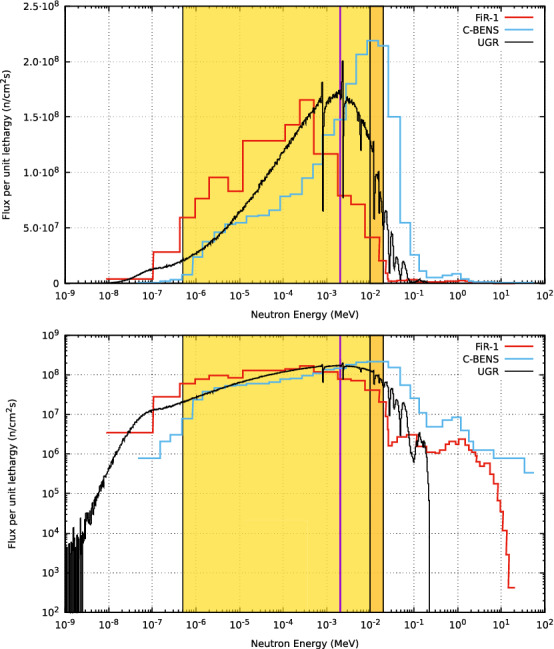
Spectrum at the BSA aperture (UGR) compared to others (FiR-1 and C-BENS) in log-lin scale (*upper panel*) and log-log scale (*lower panel*). The epithermal range from 0.5 eV to 10 keV is marked in gold and the extension from 10 to 20 keV in pale orange. The 2 keV energy is marked with a vertical dotted line.

In the following, let us overview the neutron spectrum obtained at the BSA aperture. Figure 5 shows the spectrum from our BSA design compared to previous reactor-based infrastructures as FiR-1, and C-BENS. The use of $\mathrm {^7Li}$ and a proton energy of 2.1 MeV generates neutrons with a maximum energy of 350 keV. The further moderation makes the neutron intensity vanish for energies above 200 keV. This is capital in the aim of reducing the fast neutron dose and its preeminence in superficial tissue. In the same way, the low energy tail of the spectrum is well suppressed, as in the other facilities. Moreover, the neutron spectrum is satisfactorily peaked at around 2–3 keV, which lies within the most suitable neutron energy range for deep-seated tumor treatments in BNCT, considered as the 2–9 keV range^21^.

**Figure 6:**
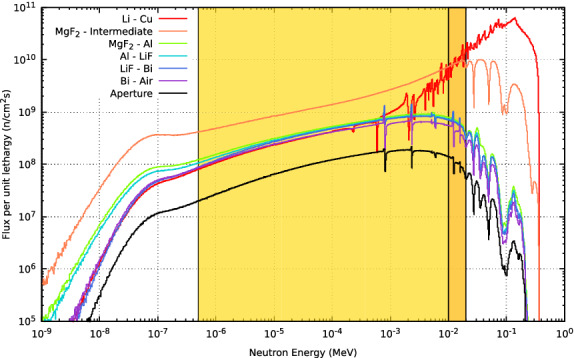
Neutron spectra at different positions of the BSA from the Lithium target to the aperture. The spectrum shaping effects from each material are exhibited. Also, an intermediate scoring plane in $\mathrm {MgF_2}$ is shown. The epithermal range from 0.5 eV to 10 keV is marked in gold and the extension from 10 to 20 keV in pale orange.

In order to go more into detail in the neutron spectrum shaping process, it is worth to mention the optimal sizing of the moderator. Figure 6 shows the progress of the moderating and beam shaping. An intermediate moderating step is registered to emphasize the need of an adequate moderating thickness. $\mathrm {MgF_2}$ is a satisfactory material in the role of moderation, productively shifting the neutron energy to the epithermal range. An increased moderator size reduces the total neutron flux available as shown when comparing the spectra at the intermediate and last $\mathrm {MgF_2}$ layers in Fig. 6. When the neutron spectrum is close to the optimal, the addition of a small layer of Al produces a relevant reduction of flux near its resonances above the epithermal range, completing definitely the moderation. The width of the Bi is reduced to the extent possible in order to limit the neutron beam degradation, but serving to attenuate the gamma contamination.

**Figure 7:**
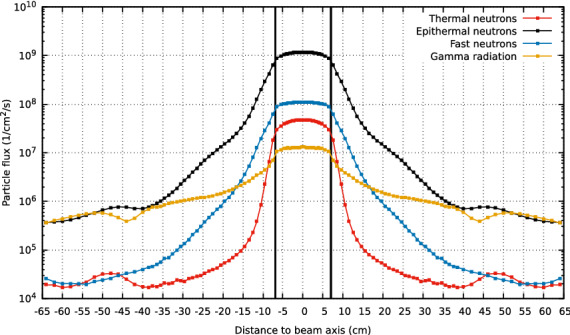
Lateral flux beam profile at the BSA aperture. Beam collimation and contamination suppression are attained.

At a more detailed level, the out-of-beam performance of the BSA is discussed below. A convenient suppression of neutrons and gamma radiation is achieved and the beam edges are well defined. The arrangement of several layers of fully-thermalizing and neutron absorbing materials together with Pb for gamma radiation attenuation effectively reduces the out-of-beam contamination. Figure 7 presents the lateral flux profiles. The neutron flux reduces by 2 orders of magnitude within the first 15 cm for epithermal neutrons, whereas this suppression is much sharper for thermal neutrons, namely 5 cm. The gamma radiation is well attenuated at safe margins throughout the entire irradiation area, even considering that off-beam suppression is less shaped compared to the neutron profiles. For instance, these improve the remarkable results of out-of-field flux profiles shown in the multiple room design at Southern Tohoku BNCT Research Center, in Japan^22^.

In order to show the front shielding performance, the neutron and gamma radiation flux distribution are displayed in Fig. 3. The neutron flux is greatly weakened outside the beam. The adjustment of the collimation angle plays a determinant role in producing a well-peaked forward beam with reduced lateral spreading off the axis. Neutrons exiting the BSA in improper directions are efficiently suppressed. Subsequent generation and shielding of gamma radiation from hydrogen captures is also noticeable.

### In-phantom Figures of Merit

After discussing the full compliance of the BSA design based on the in-air IAEA recommendations, we will focus on its clinical adequacy for BNCT treatments in relation to in-phantom dose simulations.

Several Figures of Merit (FOM) have been used to characterize the quality of the beam: Advantage Depth (AD), the depth where the dose to the tumor equals the maximum dose to the normal tissue; Advantage Depth Dose Rate (ADDR) is the maximum delivered dose rate to normal tissue; Treatable Depth (TD) defines the depth where the tumor dose falls below twice of the maximum dose to normal tissue; Maximum Treatment dose Ratio (TR) is the maximum ratio between the maximum delivered dose rate to normal and tumor tissue; Treatment time (TT) can be estimated as the time to reach the maximum allowable dose to the healthy tissue, namely 12.5 Gy; Average treatment dose Ratio (AR) is the ratio between the total tumor and normal tissue dose, each one integrated from the tissue surface to the AD.

**Figure 8:**
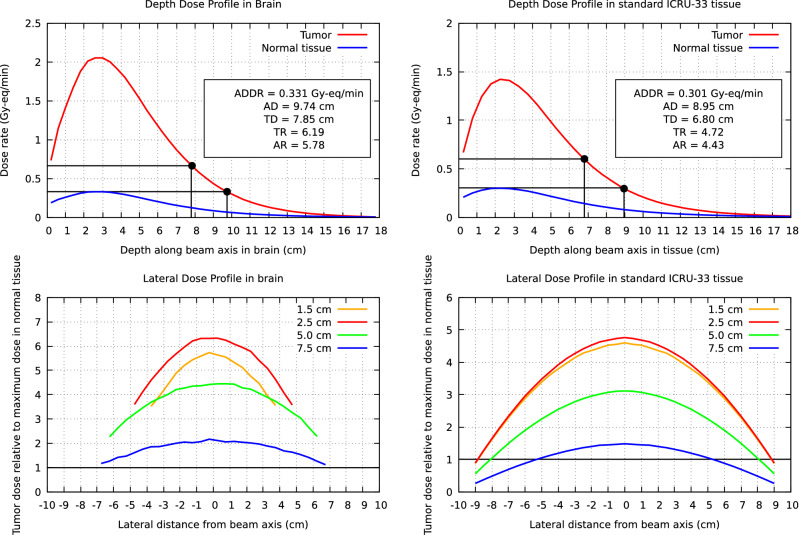
Dose profiles for brain (Snyder head model, *left*) and for standard ICRU33 tissue in a cylindrical phantom (*right*). The *upper panels* show the in-depth dose rate profiles along the beam axis. These panels include the relevant FOMs for each case, namely ADDR, AD, TD, TR and AR. The *lower panels* show the lateral dose distributions (normalized to the maximum dose in normal tissue) at four depths, namely 1.5, 2.5, 5.0 and 7.5 cm.

The depth and lateral dose profiles for a Snyder head phantom and a phantom of standard 4-component ICRU (ICRU33) soft tissue^23^ are shown in Fig. 8. The most relevant FOMs are also included in the Figure. For Snyder head phantom, AD is 9.74 cm with ADDR of 0.331 Gy-Eq/min; TD is 7.85 cm; The TR and AR ratios reach the values of 6.19 and 5.78, respectively. For standard tissue ICRU33 phantom, AD reaches the 8.95 cm, with ADDR of 0.301 Gy-Eq/min; TD is 6.80 cm; Accordingly, TR and AR ratios are 4.72 and 4.43. In both cases the maximum TT is less than 1 h, with 38 min for brain and 42 min for standard ICRU33 tissue, approximately. It is important to note that the *real* clinical treatment time can be greatly reduced taking into account the optimal tumor dose^24^ and depending of the tumor depth location.

Analyzing the reactor-based BNCT facilities as FiR-1, THOR and the Studsvik’s R2-0 in Sweden^25^, in all cases TD is less or around 7 cm, In our case, TD reaches almost 8 cm. Regarding AD, our result is one of the best among those mentioned, reaching 9.74 cm in contrast to the closest one of 9.7 cm of the Studsvik R2-0 reactor. The ADDR is 0.331 Gy/min in our case, compared with 0.45 Gy/min at FiR-1 and 0.50 Gy/min at THOR in compliance with the total neutron flux at each facility. These results highlight the therapeutic goodness of the beam, remarkably for deep-seated tumors.

Moving forward to KURRI we will compare our results with the reactor-based KUR-HWNIF and C-BENS neutron sources. For both facilities, the maximum AD value ($\simeq 10$ cm) is only achieved when assuming tumor:normal tissue ratio of 4.5 and 50 ppm boron concentration^24^. We achieve almost the same AD (9.74 cm) with a lower boron concentration ratio and less fast neutron contamination^14^.

Focusing on accelerator-based facilities, different projects have been in development in California (USA)^26^, Obninsk (Russia)^18^, Korea^27^ or Argentina^28^. None of them achieve AD higher than 9.5 cm, in comparison with our result (9.74 cm). The most recent work in Osaka (Japan)^15^ has good results in TT (24.5 min), although with AD of 9.1 cm. Another proposal was made in Novosibirsk (Russia)^19^, with similar AD (9.7 cm) result, but with less TD (7.52 cm) and TR (5.38).

It is worth to mention that in the previous comparisons, not in all cases the parameters reported from the different designs are obtained by using the same evaluation standard than in this work. In the design of the R2-0 at Studsvik, there is a difference in boron concentration, where 25 ppm of B were considered instead of the 18 ppm used at brain phantoms in this study. In the THOR beam design, the in-phantom parameters are obtained for a head located at 10 cm from the beam exit. Therefore, these comparisons have to be considered with caution. Also, we mention that the comparisons have been performed with published data, that in some cases are old. New updated data of some reactor beams could affect those comparisons.

**Figure 9:**
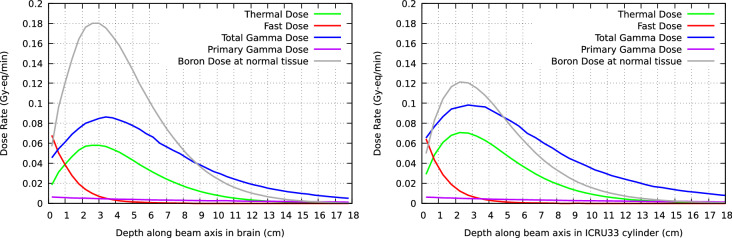
Dose rate components at the brain tissue for a model irradiation of the Snyder phantom (*left panel*) and for the ICRU33 standard tissue cylinder (*right panel*). Thermal, fast and total gamma dose rates are given. The primary gamma dose, directly impinging to the phantom from the beam is also shown separately. This remarks the low gamma contamination of the designed beam.

The contribution of the different dose components in normal brain and soft tissue are illustrated in Fig. 9. In addition to the total gamma dose (which includes the primary gammas from the beam and those produced by neutron captures by hydrogen), the contribution from the beam contamination, remarkably low, has been explicitly displayed.

**Figure 10:**
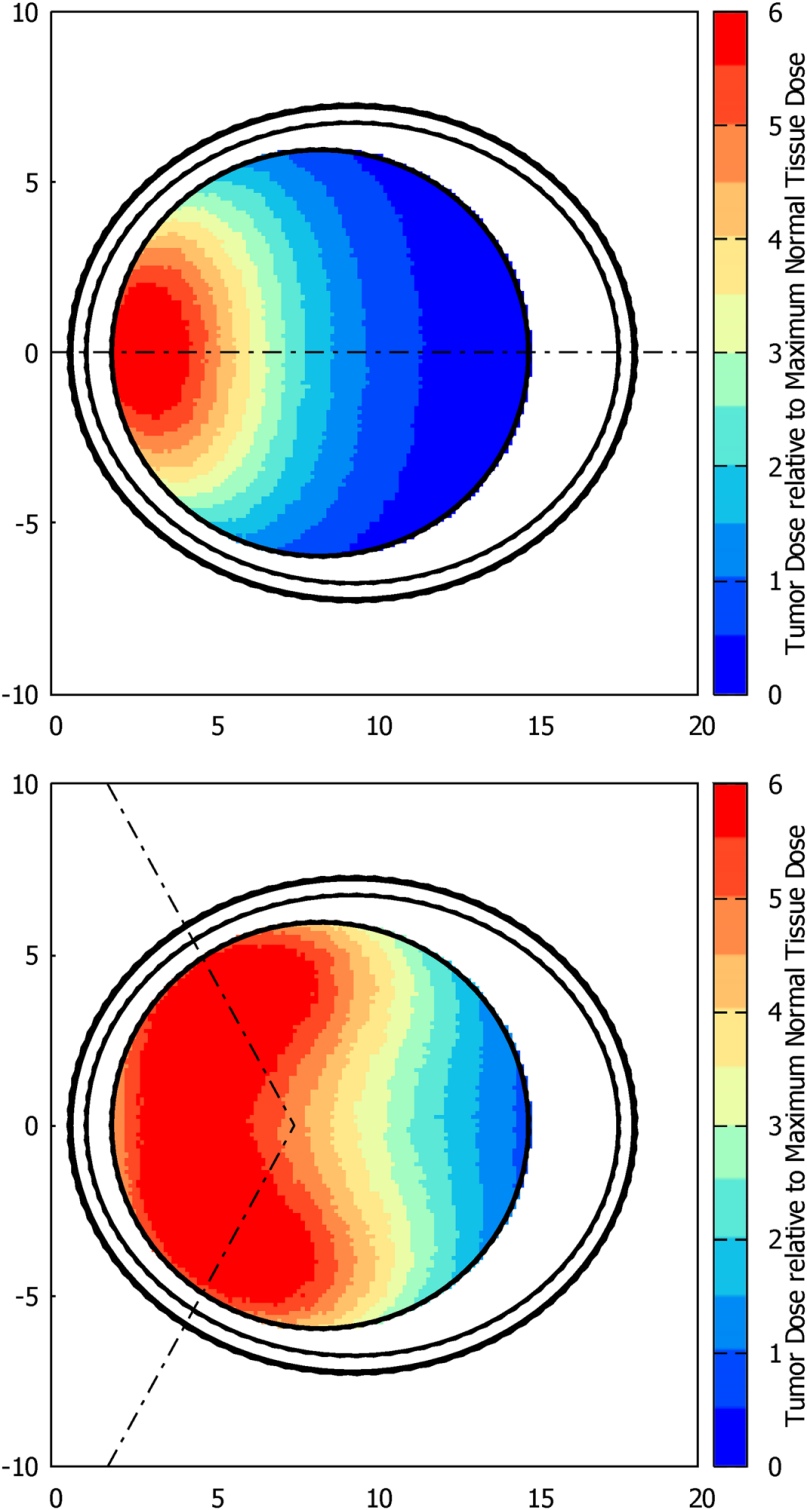
Dose map at brain (Snyder head phantom) for two irradiation procedures, with one and two fields. The one-field irradiation is frontal, the fields for the two-field irradiation are symmetrically rotated by $60^{\circ }$ each with respect to the head axial symmetry axis. Dashed-dotted lines indicate the beam axis for each irradiation field. The color scale is normalized to the maximum dose in normal tissue.

Finally, just in order to illustrate the capabilities of the beam to cover a wide volume of tissue for BNCT therapies, an example of a one-field and a two-field irradiation are shown in Fig. 10. The color map illustrates the equivalent dose at tumor relative to the maximum dose in normal brain tissue. The maximum dose rate in normal tissues, delivered at scalp, even considering the higher data for the boron concentration (1.5 times the concentration in brain) and CBE at skin (2.5), is 0.36 Gy-eq/min, close to the maximum dose delivered at normal brain (0.331 Gy-eq/min), for the single beam case. As the tolerable dose is much higher (24 Gy-eq. respect to 12.5 Gy-eq for brain), the skin dose is not a problem. The dose at skull is much smaller. As one could notice, a simplistic two-field irradiation almost covers the full brain region, with a tumor- to-normal dose ratio greater than 2. Although none corresponds to an actual treatment, it suggests that a *multi*-field irradiation with a desirable angle-positioning combination may reach any tumor location in brain with a therapeutic dose.

## Conclusions

A design of a beam shaping assembly for an accelerator-based BNCT facility has been presented. It improves in terms of both in-air and in-phantom figures of merit compared to previously proposed devices. The well defined neutron beam allows a safe clinical operation within a hospital, and as a novelty, requires a low energy proton accelerator (up to 2.1 MeV). The features of this design allow, according to Monte Carlo simulations, to treat tumors with one or two beams located in a wide range of tissue, in particular, for brain tumors in any part to the brain, provided the tumor uptake of the boron compound.

It is worth to mention that the physics of the neutron production and moderation at these proton energies (below the inelastic threshold) are very well known which guarantees that the Monte Carlo simulations will give a realistic estimation of the performance of this device.

## Methods

### Beam shaping assembly design

In the following, the process of designing the BSA is described.

The $^7Li(p,n)^7Be$ thick-target yield near the reaction threshold was computed according to Lee and Zhou^29^.

The process to determine the most appropriate materials and sizes was based on the search for isotopes where the elastic interaction dominates.

The presence of few resonances above the epithermal range was considered as a key factor. In addition, mean-free-path and moderation estimations were computed to estimate the sizes of the main components. Once this process derived in a reasonable solution, an optimization based on perturbing all the dimensions and sizes was followed. That procedure kept ongoing until a good compromise was reached among the best suited sizes for all IAEA recommendations, in-phantom FOMs and beam shaping needs.

The neutron transport simulations were carried out using the Monte Carlo code MCNP6^30^.

Independent simulations were run to determine also the influence of the gamma radiation production at $\mathrm {^{7}Li}$ in the total gamma contamination at the beam aperture. An isotropic, 478 keV photon source was used, whose yield was normalized with respect to the neutron production following the tabulation at Lee^31^. In addition, gamma emission from activation was evaluated in the case of $\mathrm {^{27}Al}$ due to the low half-life of $\mathrm {^{28}Al}$ as in previous studies^26^. An upper bound for delayed gamma emission from decays was included in the gamma dose contamination computations via these MCNP6 simulations.

### Analysis of the beam properties

The fast neutron and gamma dose contamination were derived integrating the flux with the corresponding kerma factors. Neutrons in the range between 0.5 eV and 10 keV are considered epithermal.

In order to evaluate the therapeutic effectiveness of the neutron beam, in-phantom dose profiles simulations were performed. A cylinder phantom of ICRU standard tissue denoted as ICRU33^23^ and the Snyder head phantom for skull and brain^32^ were used.

The total photon-equivalent dose (Eq. 1), in grays-equivalent (Gy.-Eq.), was estimated as separate contributions of thermal neutron dose ($\mathrm {D_t}$), fast neutron dose ($\mathrm {D_f}$), boron ($\mathrm {D_B}$) and gamma dose ($\mathrm {D_\gamma }$), weighted to each compound factor (CP).

1 $$\begin{aligned} D_{T} = w_t \cdot D_{t}+ w_f \cdot D_{f} + w_B \cdot D_{B} + w_{\gamma } \cdot D_{\gamma } \end{aligned}$$

The weighting factors reflect the relative biological effectiveness (RBE) of each component, with the following values: 3.2 for thermal and fast neutrons; 3.8 and 1.3 for boron contribution in tumor and normal tissue, respectively; 1 for gamma contribution^33^. The tumor and normal tissue $\mathrm {^{10}B}$ concentration for standard tissue (ICRU33) was set at 35 and 10 ppm, respectively. It has been reported that for the most severe brain tumors (i.e. glioblastoma) the $\mathrm {^{10}B}$ concentration ratio (tumor:normal) is greater than 3.5:1^34,35^. Therefore, in Snyder phantom the $\mathrm {^{10}B}$ concentrations were assumed to be 65 and 18 ppm for tumor and normal brain tissue, as in previous studies^13,18,36^.
